# Supercapacitors and triboelectric nanogenerators based on electrodes of greener iron nanoparticles/carbon nanotubes composites

**DOI:** 10.1038/s41598-024-61173-5

**Published:** 2024-05-21

**Authors:** Glaydson Simoes dos Reis, Helinando Pequeno de Oliveira, Iuri Custodio Montes Candido, Andre Luiz Freire, Palanivel Molaiyan, Guilherme Luiz Dotto, Alejandro Grimm, Jyri-Pekka Mikkola

**Affiliations:** 1 Department of Forest Biomaterials and Technology, Biomass Technology Centre, Swedish University of Agricultural Sciences, 901 83 Umeå, Sweden; 2 Institute of Materials Science, Federal University of Sao Francisco Valley, Petrolina, 56304-205 Brazil; 3 Research Unit of Sustainable Chemistry, University of Oulu, P.O. Box 3000, 90014 Oulu, Finland; 4 Research Group On Adsorptive and Catalytic Process Engineering (ENGEPAC), Federal University of Santa Maria, Av. Roraima, 1000-7, Santa Maria, RS 97105–900 Brazil; 5 Technical Chemistry, Department of Chemistry, Umeå University, 90187 Umeå, Sweden; 6 Industrial Chemistry and Reaction Engineering, Johan Gadolin Process Chemistry Centre, Åbo Akademi University, 20500 Åbo-Turku, Finland

**Keywords:** Green synthesis, Iron nanoparticles, Flexible electrodes, Energy harvesting and storage, Energy science and technology, Materials science

## Abstract

The development of supporting materials based on carbon nanotubes (CNTs) impregnated with iron nanoparticles via a sustainable and green synthesis employing plant extract of Punica granatum *L.* leaves was carried out for the iron nanoparticle modification and the following impregnation into the carbon nanotubes composites (CNT-Fe) that were also coated with polypyrrole (CNT-Fe + PPy) for use as electrode for supercapacitor and triboelectric nanogenerators. The electrochemical characterization of the materials by cyclic voltammetry (CV) and galvanostatic charge–discharge (GCD) assays revealed that the CNT-Fe + PPy gave rise to better performance due to the association of double-layer capacitance behavior of carbon derivative in association with the pseudocapacitance contribution of PPy resulting in an areal capacitance value 202 mF/ cm^2^ for the overall composite. In terms of the application of electrodes in triboelectric nanogenerators, the best performance for the composite of CNT-Fe + PPy was 60 V for output voltage and power density of 6 μW/cm^2^. The integrated system showed that the supercapacitors can be charged directly by the nanogenerator from 0 to 42 mV in 300 s. The successful green synthesis of iron nanoparticles on CNT and further PPy coating provides a feasible method for the design and synthesis of high-performance SCs and TENGs electrode materials. This work provides a systematic approach that moves the research front forward by generating data that underpins further research in self-powered electronic devices.

## Introduction

The fast depletion of fossil fuels and environmental pollution are driving forces that motivate the research on renewable and green energy sources. Electrochemical devices for energy storage such as supercapacitors (SCs) and batteries, and the harvesting of energy such as triboelectric nanogenerators (TENGs) have been widely employed in portable and wearable electronic devices. The storage and release of energy in SCs are governed by the electrochemical interactions between electrolytes (ions) and electrodes^1–3^ being considered powerful devices with fast charging/discharging, large power density, and long cyclability, which render them very suitable for integration with energy-harvesting devices such as TENGs, which have been extensively explored as promising templates to harvest energy from mechanical movement^4^. An alternative that has been recently explored for TENGs is the association with magnetic nanoparticles. The incorporation of magnetic domains in friction layers is an additional source for repulsive forces in substitution to conventional mechanic restoring forces and in the induction of electric potential in copper-based electrodes^5^. Several applications have been explored for these materials such as the use in tilt sensing systems, noncontact mode TENG by the action of an external magnetic field^6^, or for magnetic field detection^7^.

The energy harvesting from TENGs is based on the contact/separation of tribopairs that takes place from the processes of contact electrification and electrostatic induction that are established at the interface between dielectric and conductive electrode material layers, acting as current collectors to give suitable current transfer to an external load resistance. TENG converts mechanical energy into electrical energy and makes possible the conversion of the body's movement into electricity. Besides, both TENGs and SCs can be integrated to create a self-charging, uninterrupted power supply system. Compensating energy consumption and extending the working duration of electronic devices’ materials have proven to be essential in the integration of TENGs and supercapacitors, providing a viable solution for developing efficient, lightweight, and flexible energy harvesting and storage devices. Both SCs and TENGs possess the advantages of lightweight, wide-ranging materials selection and simple fabrication process, being considered as a promising energy source for wearable electronics such as smart bracelets^8^, smart textiles and clothing^9,10^, and flexible displays^11^. The high performance of the SCs strongly relies on the electrode properties making necessary the research for efficient active electrode materials for high-capacity SCs^12,13^. Overall, SC electrodes are commonly composed of carbon-based materials^14,15^, transition metal composite materials^16^, and polymer-based materials^17^.

Some of these compounds such as metals and their respective oxides present both high conductivity and capacitance, however, they face poor electrochemical reversibility, and poor energy harvesting behavior due to their unsuitable elastic properties, which hinders their efficient application in stable and high-performance SCs^18^ and TENGs^19^. On the other hand, carbon materials including carbon cloth, graphene, and carbon nanotubes are widely employed as suitable electrodes for SC and TENG applications due to their excellent conductivity, chemical stability, and resistance to corrosion^14,20^.

For instance, carbon nanotubes (CNTs) exhibit important advantages for application in electrodes for SCs and TENGs, such as high specific surface area, high density, outstanding conductivity and chemical stability, and unique hollow and layered structure, which facilitate ion transport; in addition, CNTs have good mechanical flexibility being considered good carriers for metal oxides^21^ which makes it very suitable for TENGs application^4,22^. However, carbon-based SCs and TENGs may face issues related to limited conductivity. A strategy to circumvent this drawback is the combination of carbon nanotubes with metal nanoparticles/conducting polymers if considering the excellent performance of pure carbon electrodes with the boosting in the electrochemical stability/performance provided by the fillers^23,24^.

Over the years, the fabrication of nanomaterials for a wide range of applications including energy harvest and storage has exponentially increased^25–27^. Several methods are employed for the synthesis of nanomaterials including chemical vapor deposition, plasma-assisted physical vapor deposition, aerosol processes, spray pyrolysis, and others^28^. However, these methods have been gradually substituted by green synthesis methods due to the serious disadvantages related to the large energy and chemical consumption, use of complex equipment and synthesis conditions^29,30^, and toxic reagents^31,32^. A sustainable and greener synthesis has been based on the combination of biomasses in conjunction with metal nanoparticles to be impregnated on the CNT structure^32,33^. This technique brings important environmental and economic gains due to the use of non-toxic, low-cost, and naturally abundant resources for metal-nanoparticles (NP) synthesis^34^, which explores the chemical reduction of the metal employing bio-based extracts from plants, fungi, bacteria sources, etc.^35^. The formation of stable metal-NPs in a support material, e.g., carbon nanotubes, can be produced by the immersion of the support material into the desired metal solution and the reducing agent extract^36^. The literature reports different bio-based materials that have been employed as reduction agents in the metal-NPs syntheses including banana peel^37^, yellow trumpetbush leaves^38^, etc. Meanwhile, common support materials are reported to be activated^39^ such as silica^40^, carbon nanotubes^41^, and graphene^42–44^. The incorporation of metal nanoparticles (e.g., iron nanoparticles) on CNT structure is an interesting approach for high-performance electrodes for SCs due to the combined/synergic effects of each material such as CNT assists in maintaining mechanical integrity and high electrical conductivity of the overall electrodes due to their superior mechanical properties, good electrochemical stability, and excellent conductivity^21,45,46^. The iron oxide nanoparticles dispersed on the CNT structure can greatly shorten the diffusion and migration paths of electrolyte ions during the rapid charge–discharge process, which assures better electrochemical properties^21,46,47^.

This work describes the synthesis of an advanced flexible electrode material for application in SCs and TENGs through a sustainable and greener approach using eggshell membrane as flexible support material and a plant-based reductant (*Punica granatum* L. Leaves) to form and deposit iron NPs on the carbon nanotube structure. The iron-NPs-CNTs were successfully applied as electrodes for SCs and TENGs. With this aim, mechanical support was provided by the eggshell membranes that were coated with iron-NPs-NT material to serve as sustainable and flexible electrodes. Further, the coated eggshell membranes with iron-NPs-NTs material were subjected to a chemical polymerization of polypyrrole (PPy), which creates a layer over the flexible electrode that preserves the electrochemical properties of additives (EDLC and PC) and the mechanical characteristics of the eggshell membrane. The flexible electrodes were fully characterized in detail to evaluate the effect of both iron-NPs-NTs and PPy on their physicochemical and electrochemical properties for both applications.

## Materials and methods

### Green synthesis route

**Figure 1:**
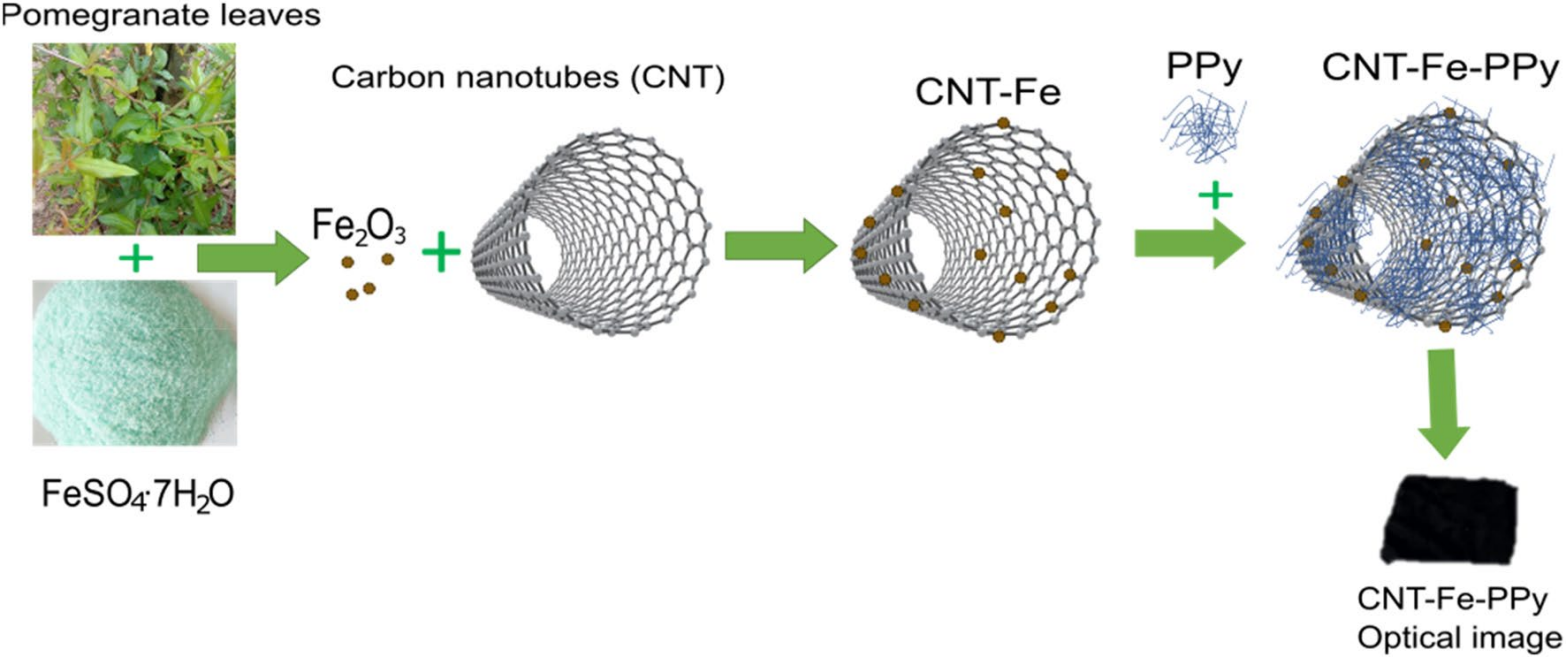
Synthesis process of the carbon electrode materials.

The commercial CNTs were impregnated with iron nanoparticles based on the method described by Wang et al*.*^32^ and its schematics are shown in Fig. 1. Extracts of pomegranate were employed as a green strategy to coat the CNTs with iron nanoparticles. Firstly, 60 g of pomegranate leaves (fallen leaves, waste biomass) were mixed with 1 L of deionized water at 80 °C and agitated for 1 h. Then, the solution was cooled, and filtrated to obtain a solid material from the vegetal extract. The preparation of the metallic solution containing iron nanoparticles was done by, first, diluting 1.5 wt% (mass volume) of iron salt (FeSO_4_·7H_2_O). After that, the solution containing 2 g of CNTs, and the salt were added and mixed with the plant extract. A ratio of 2:1 in volume (40 mL of extract and 20 mL of salt solution) was used. The solution containing all compounds was agitated (160 rpm) using a stirrer at 25 °C and for 24 h. As a final step, the solid materials were separated through filtrations and dried at 105 °C for 24 h for further use.

### Removal and treatment of eggshell membranes (ESM)

The eggshells were obtained from the local market in the city of Juazeiro-BA, Brazil, and the cleaning and membrane extraction procedure was carried out according to the procedure described by Alcaraz-Espinoza et al*.*^48^ According to the reported procedure, the eggshells were disposed of in 1 M HCl solution for 30 min. Subsequently, the eggshell membranes (ESM) were separated from the shell and washed several times with ultrapure, deionized water (Milli-Q water). Then, the samples were cut into 8 mm diameter discs for further use.

### Electrode preparation

Three samples, viz*.* Fe + CNT, PPy, and CNT-Fe + PPy were prepared to evaluate the influence of components in the overall response of the modified eggshell membranes applied as electrodes of the electrochemical devices. The preparation steps are described as follows: The synthesis of sample PPy provided the free growth of polypyrrole on the unmodified eggshell membrane that was conducted according to the procedure reported in Ref.^49^: two solutions (S1 and S2) were prepared. The solution S1 was prepared with 25 mL of HCl (1 M) in which pyrrole (35 μL) was added to a beaker and stirred for 5 min. Subsequently, the membrane was added and the solution was stirred for 30 min in an orbital shaker at room temperature. For the preparation of the solution S2, 81.1 mg of FeCl_3_ was added to 25 mL of HCl (1 M) and kept under intense stirring. As a following step, the solution S2 was slowly dropped into S1 and stirred for 24 h on the orbital shaker, at room temperature. After that, the membrane was removed washed with ultrapure water, and dried at room temperature. After drying, a medium weight for the PPy sample transferred to ESM of 4.7 mg was obtained—thickness of 74 µm. Disks of 8 mm in diameter were applied as electrodes for supercapacitors while samples of 2 cm × 3 cm were applied in the harvesting of energy as electrodes of TENGs. Photos of the resulting electrodes are shown in Fig. S1.

A mother solution containing 10 mg/mL of Fe + CNT aqueous solution was used to transfer under sonication the modified carbon nanotubes to the ESM. After two cycles of sonication of membranes into Fe + CNT solution of 45 min, the medium of mass for adhered nanostructures is 2.5 mg—thickness of 79.5 µm. The sample CNT-Fe +/PPy is prepared by the combination of two steps in which an initial deposition of Fe + CNT (as previously described) is followed by an additional step of polymerization of PPy (as described for sample PPy). Under the combination of these steps, the final weight of the impregnated material was in the order of 6.7 mg—thickness of 78 µm.

## Results and discussion

### Morphological and structural characterization

**Figure 2:**
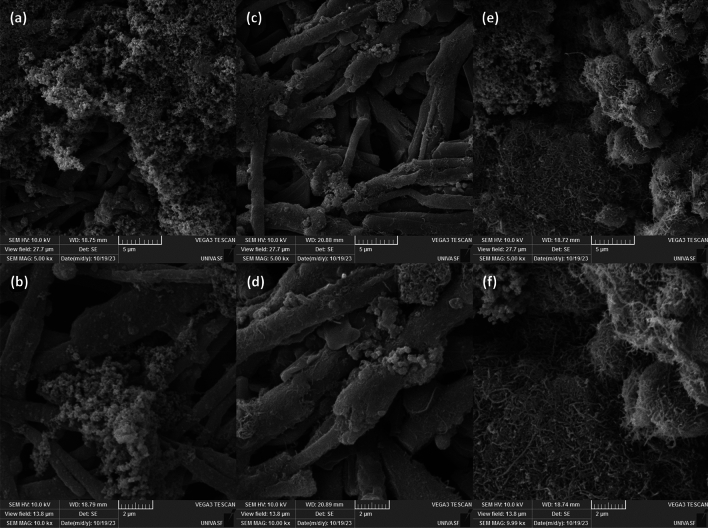
Morphology of modified ESM templates at different magnifications (**a**, **b**) for PPy, (**c**, **d**) CNT-Fe, and (**e**, **f**) for CNT-Fe + PPy.

The morphology of PPy (Fig. 2a,b), CNT-Fe (Fig. 2c,d), and CNT-Fe + PPy (Fig. 2e,f) deposited on ESM were evaluated from SEM images. As can be observed, the overall morphology of the ESM supports is composed of a distribution of tubular structures with different diameters and 3D micrometer sizes; the overlapping of CNTs seems to create random voids and spaces (yielding porous structures). The CNT-Fe + PPy structure showed a much different morphology with the disappearance of 3D tubular structures and bigger voids, but the resulting morphology with agglomerates was a coherent surface feature, indicating a highly stable PPy loading and good compatibility. It can be stated that the CNT-Fe + PPy electrode surface demonstrated a 3D-fine porous stacking formation, with a network of conducting PPy structure. CNT-Fe + PPy can be characterized as a layered growth of nanofoam features over the CNT-Fe structure. It is expected for the CNT-Fe + PPy system to a better conductivity and electrochemical performances when tested as flexible SCs. Furthermore, EDS images are shown in Fig. S2, which confirms the presence of nitrogen and iron elements, with the presence of the N justified by the coating with PPy, confirming the adequate modification with PPy.

The pore structure characteristics without and under doping with the PPy on ESM + CNT-Fe were also evaluated. The results show that after PPy doping the sample-specific surface area decreased from 68 m^2^ g^−1^ (CNT-Fe) to 14 m^2^ g^−1^ (CNT-Fe + PPy). Both samples exhibited a more pronounced presence of mesopores (see Table 1). These results agree with the data reported in the literature^50,51^. The SSA decrease is due to the deposition of PPy over the CNT-Fe surface covering the pores and filling the voids of the CNT-Fe agglomerates.

**Table 1:** CNT-Fe and CNT-Fe + PPy surface area properties.

	SSA	A_Micro_	A_Meso_	Pore volume
	(m^2^ g^−1^)			(cm^2^ g^−1^)
CNT-Fe	68	22	46	0.46
CNT-Fe + PPy	12	3.4	8.6	0.092

**Figure 3:**
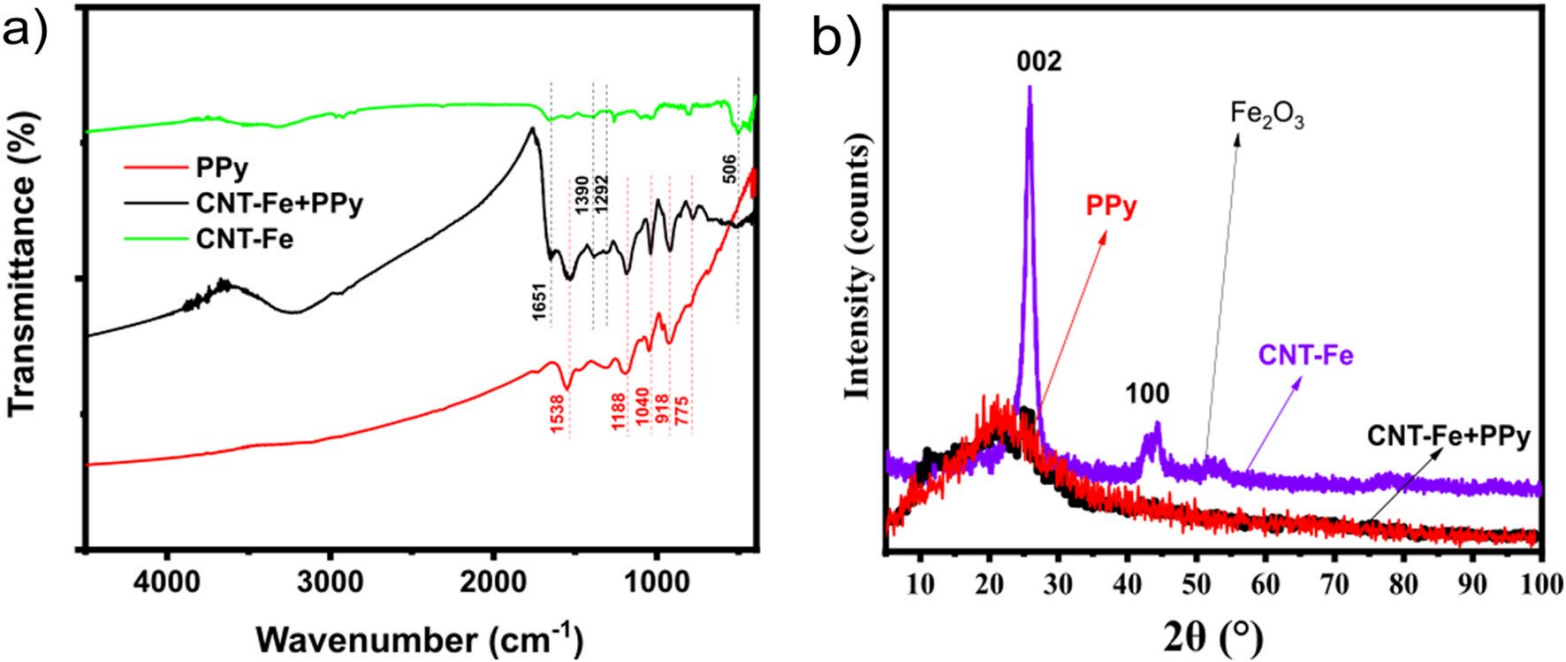
(**a**) FTIR spectra of the PPy, CNT-Fe, and CNT-Fe + PPy electrodes, and (**b**) XRD patterns of the PPy, CNT-Fe, and CNT-Fe + PPy electrodes.

The FTIR analysis (Fig. 3a) of the PPy and CNT-Fe + PPy electrodes gives valuable information about the surface chemical characteristics regarding the presence of surface groups induced by the polymerization of polypyrrole on ESM and CNT-Fe structure. For the CNT-Fe + PPy sample, the presence of absorption bands at 1651, 1536, 1390, 1188, 1040, and 775 cm^−1^ was observed, which is by the PPy data reported in literature^52^. In comparison with the PPy spectrum, it is possible to confirm the presence of peaks at 1651, 775, and 3440 cm^−1^, respectively, are attributed to the C–N, and N–H stretching vibration in the PPy ring^53^. The above data suggest that the PPy was successfully incorporated into the CNT-Fe sample, which could implicate improved electrochemical/energy performances when used as electrodes for SCs and TENGs, respectively^50,54^.

The microcrystalline and amorphous structure of the CNT-Fe and CNT-Fe + PPy samples were evaluated by using wide-angle X-ray diffraction (Fig. 3b). The XRD patterns of CNT-Fe shows one broad peak assigned to the (002) lattices of hexagonal graphite have appeared at 2θ = 25.8°; a low-intensity peak appeared at 43.2°, corresponding to diffractions of the graphitic planes (100). A small diffraction line at 2θ of 50.9° may correspond to Fe_3_O_4_ (JCPDS No 19-0629) is identified in CNT-Fe^55^. The PPy sample exhibited a broad diffraction peak (amorphous nature) of PPy between 10° and 26° characteristic of an amorphous structure of polypyrrole^56,57^. The XRD patterns CNT-Fe + PPy exhibited a similar amorphous nature of PPy between 10° and 26° characteristic due to the influence of polypyrrole. These patterns are similar to the literature's results regarding carbon nanotubes doped with PPy^56,57^. These data indicate that green synthesis did not affect the crystallinity of carbon nanotubes, preserving their structure while the incorporation of PPy changed to a completely amorphous structure.

### Electrochemical performance of CNT-Fe and CNT-Fe + PPy based supercapacitor

**Figure 4:**
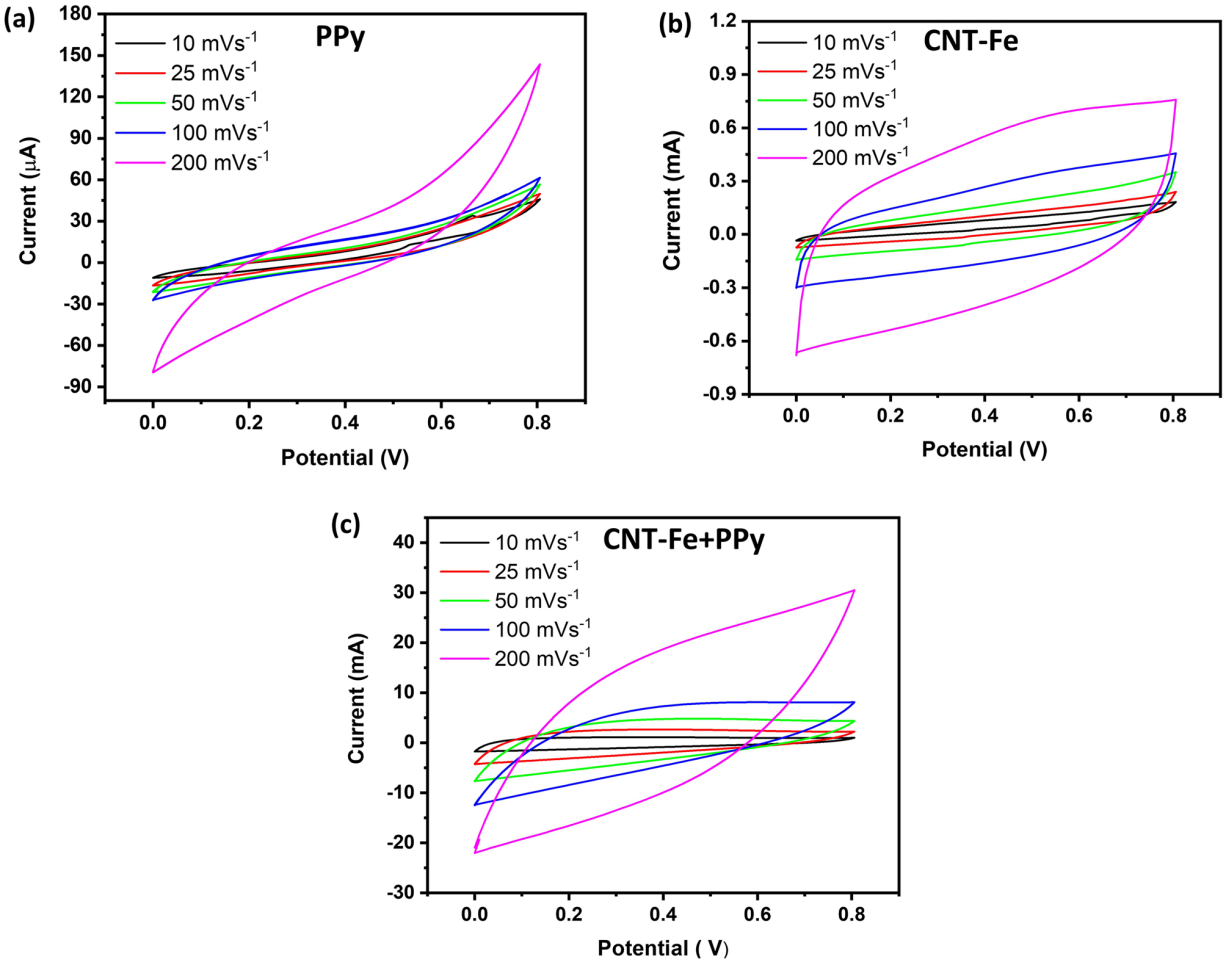
CV curves at different scan rates for SCs composed with PPy, CNT-Fe, and CNT-Fe + PPy samples.

The pure PPy, CNT-Fe, and CNT-Fe + PPy samples were integrated into symmetric supercapacitors utilizing a KOH electrolyte (1 M) impregnated into a Celgard membrane separator to assess the electrochemical performance at scan rates ranging from 10 to 200 mV s^−1^ (see Fig. 4a–c). The curves displayed distinct behaviors, thus evidencing the role of the PPy on the electrochemical performance of the SCs. For both CNT-Fe and CNT-Fe + PPy systems, a quasi-rectangular behavior was more evident in CNT-Fe (in special at a low scan rate) while the curve for the PPy-based SC appeared as a more cone-shaped, even at a low scan rate. With the increase of the scan rate values, the curve format acquired a more prolate behavior, with a cone-shaped response at a high limit (200 mVs^−1^) for both CNT-Fe and CNT-Fe + PPy, SCs. Pure PPy exhibited a completely distorted form of the CVs with very low areas compared to the CNT-Fe and CNT-Fe + PPy SCs, respectively because of the absence of EDLC contribution from a carbonaceous derivative. It is worth mentioning that the area enclosed by the CV curves is related to the specific capacitance of the studied SCs^50^. A bigger CV curve area indicates better electrochemical performance and, therefore, more efficient SCs. In this sense, CNT-Fe + PPy displayed higher areas of the CV curves under the selected scan rates, suggesting that the incorporation of PPy on previously deposited CNT + Fe introduced the SCs changes possibly due to the pseudocapacitive behavior of the PPy that led to an increase in the areal or gravimetric capacitance. The largest CV area was found to be of the CNT-Fe + PPy SCs, which indicates its best capacitive performance. The comparison of samples at the same scan rate (100 mVs^−1^) is provided in Fig. S3.

**Figure 5:**
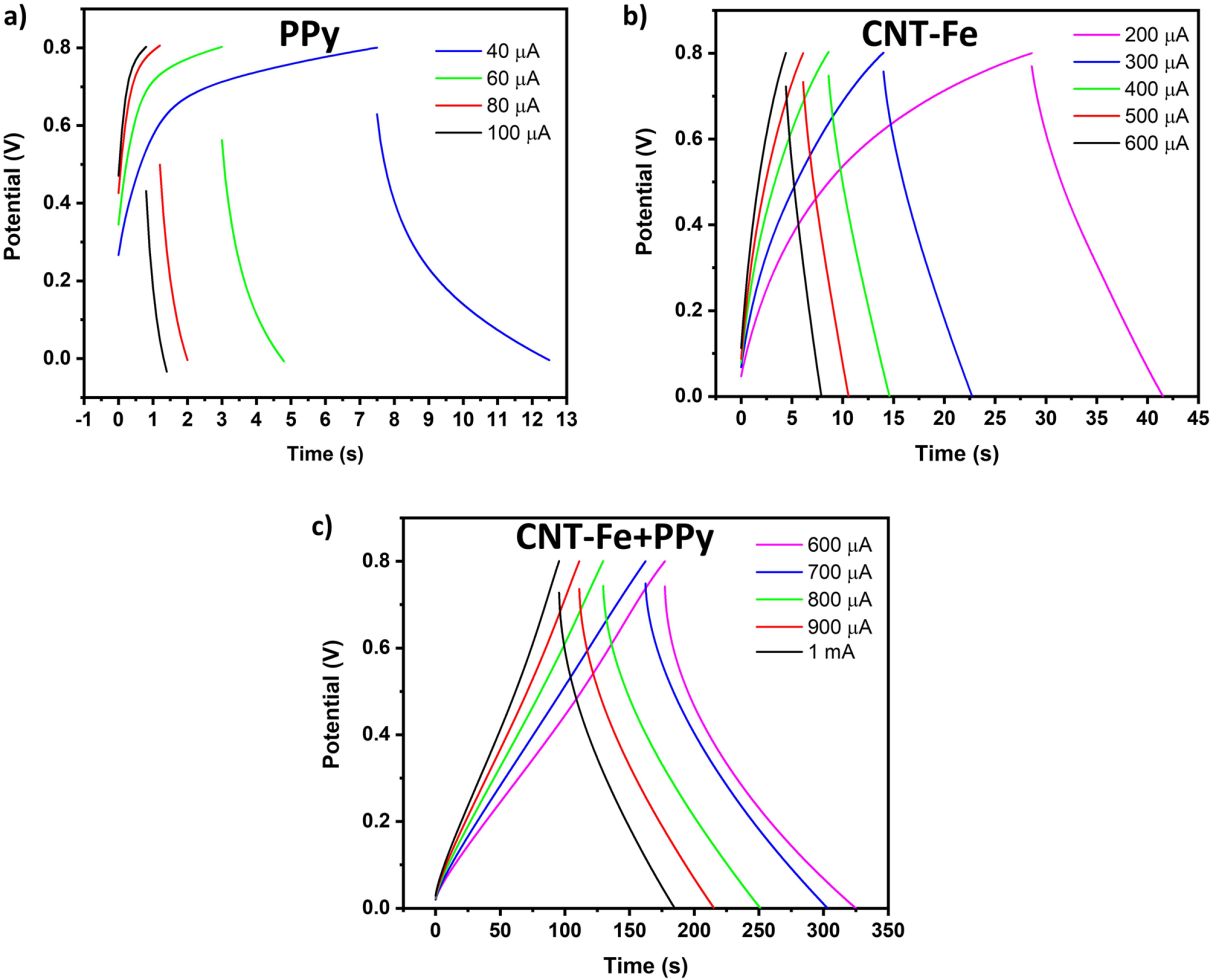
Galvanostatic charge–discharge (GCD) curves at various current densities for SCs composed with PPy, CNT-Fe, and CNT-Fe + PPy samples.

The electrochemical performance of the symmetric SCs was further evaluated by galvanostatic charge–discharge (GCD) curves, at different current densities (see Fig. 5a–c, respectively). The results show that pure PPy gave rise to very unstable GCD curves with a much shorter time compared to CNT-Fe and PPy + CNT-Fe SCs (see Fig. 5a–c, respectively). The discharge time of the symmetric SCs supercapacitor assembled by CNT-Fe + PPy was nearly 150 s and 90 s at 600 µA and 1 mA, respectively, which was much longer than that of CNT-Fe, indicating its excellent energy storage characteristics due to the PPy loading^58^. Such results agree with the observed CV profiles: The better electrochemical performance of CNT-Fe + PPy is suggested to be caused by prolonged time in the GCD curves. This improvement behavior of the PPy was already reported by Dos Reis et al.^51^ that coated a biomass-derived activated carbon with PPy. Further, the evident deviation from the ideal straight line in the GCD curves is due to Faradaic (pseudocapacitive) mechanisms.

**Figure 6:**
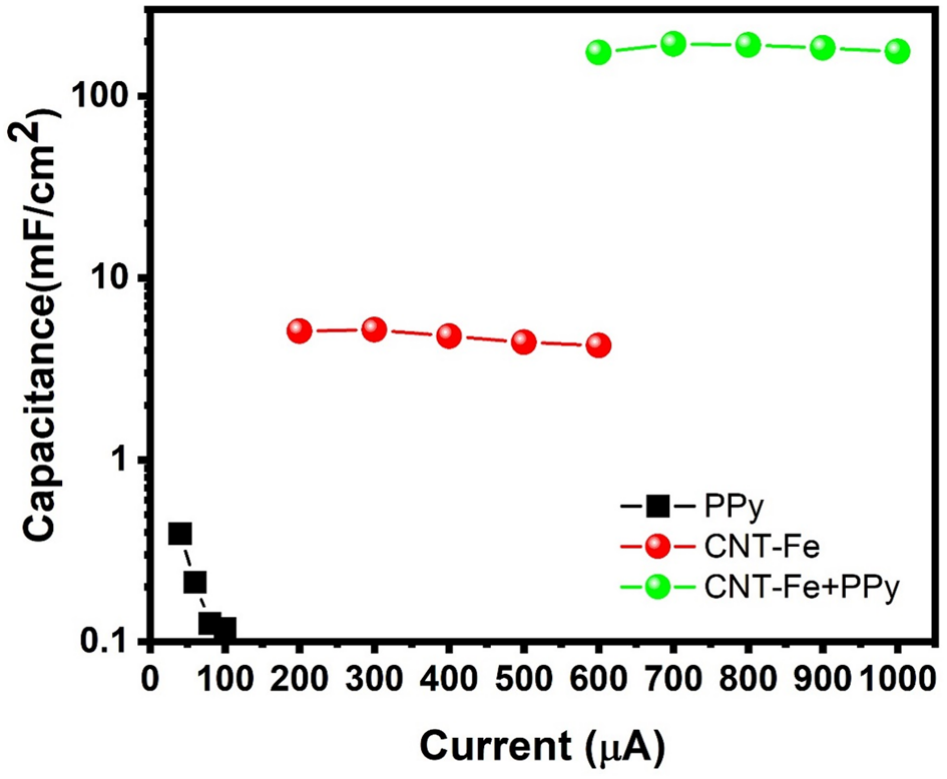
Areal capacitance values for the three SCs systems.

The areal capacitances for the three SCs systems (PPy, CNT-Fe, d CNT-Fe + PPy) were calculated from the area below the discharge curve (C_A_ = $\frac{\int idt}{A\Delta V}$) where A is the area of electrodes and ∆V is the voltage window. The areal capacitance for PPy SCs was extremely low—less than 1 mF cm^−2^—and CNT-Fe delivered an areal capacitance of around 7 mF cm^−2^. However, when CNT-Fe was coated with PPy, the SCs delivered a very high areal capacitance of 202 mF cm^−2^ and it remained stable for all the current densities (see Fig. 6). This indicates that the PPy coating on the CNTs surface was vital to improve the electrochemical performance of the CNT-Fe + PPy electrode. The areal capacitance of various electrode materials for SCs is depicted in Table 2. The areal capacitance of CNT-Fe + PPy is among the highest values, which demonstrates its feasibility as an efficient electrode for supercapacitor applications.

**Table 2:** The areal capacitances of different electrode materials for supercapacitors.

Electrode material	Areal capacitance	Electrolyte	Current density	Reference
Raw Pleurotus eryngii	4.6 F cm^−2^	6 M KOH	10 mA cm^−2^	^59^
Bamboo shells	1.53 F cm^−2^	1 mol L^−1^ H_2_SO_4_	1 A g^−1^	^60^
Bacterial cellulose	2106 mF cm^−2^	6 M KOH	10 mA cm^−2^	^61^
Soybean root	3.5 F cm^−2^	EMIM BF_4_ or 6 M KOH	1 A g^−1^	^62^
Wheat bran	2.8 F cm^−2^	1 M Na_2_SO_4_	0.5 A g^−1^	^63^
Coffee grounds	3.9 F cm^−2^	6 M KOH	1.2 mA cm^−2^	^64^
PPy	1 mF cm^−2^	KOH	1 mA g^−1^	This work
CNT-Fe	7 mF cm^−2^	KOH	1 mA g^−1^	This work
CNT-Fe + PPy	202 mF cm^−2^	KOH	1 mA g^−1^	This work

**Figure 7:**
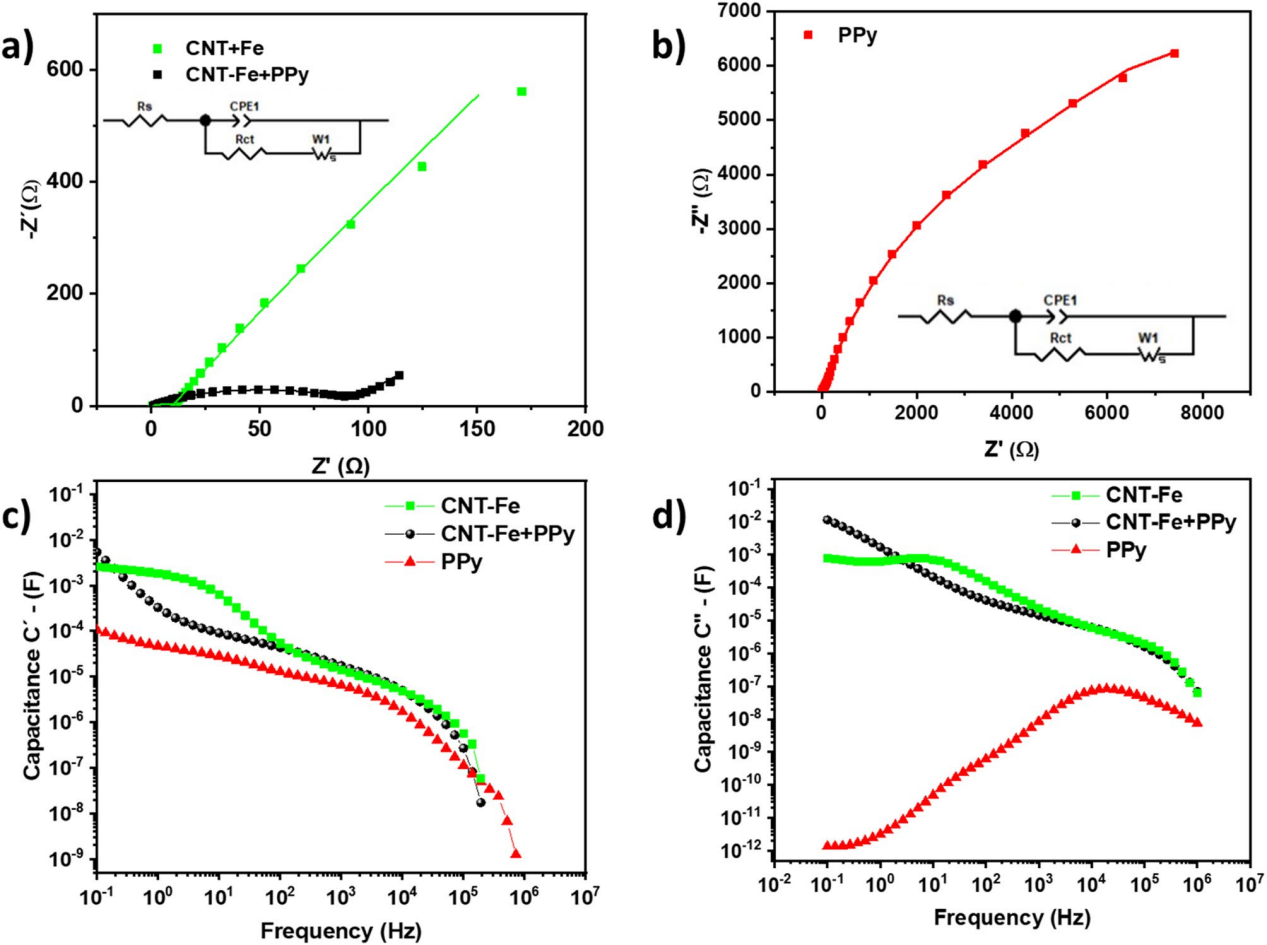
(**a**) Nyquist diagram of CNT-Fe and CNT-Fe + PPy SCs (0.01 Hz–1 MHz), (**b**) Nyquist diagram PPy (0.01 Hz–1 MHz), (**c**) real part of areal capacitance versus frequency, and (**d**) imaginary part of capacitance versus frequency.

The Nyquist plot’s semi-circle identification is a crucial parameter since it facilitates the understanding of the key limitation of the interface resistance, which enables a proper SC optimization^65^. Minimizing resistance and maximizing capacitance is extremely important to fabricate high-performance electrodes for SC applications^65^. The capacitive features of the SCs’ samples are further evaluated taking into consideration the relative variation in the linear line slope at a low frequency, while the intersection with the *x*-axis gives the estimated value for the internal resistance. Nyquist plots (shown in Fig. 7) were evaluated to provide additional information about the outstanding performance observed for CNT-Fe+ PPy samples. The curves reveal the relevance and the impact of the PPy on the electrochemical properties of our CNT-Fe electrode. Nyquist plot of CNT-Fe (curve in red in Fig. 7a) reaches resistance in the order of 600 Ω at a low frequency while the corresponding system prepared with the coating of PPy reduces this value to 100  Ω. On the other hand, an extremely high resistance is obtained for electrodes of PPy, justifying the low performance for PPy-based supercapacitors, in clear evidence that a previous deposition of a conductive layer is critical to improve the pseudocapacitance of polypyrrole (more homogenous deposition with minimal formation of aggregates of grains, as shown in SEM images for CNT-Fe + PPy).

To quantitatively evaluate the EIS of the three electrode materials, the overall spectrum was fitted by an equivalent circuit (inset of Fig. 7a), the Randles circuit, which shows the straight line in the low frequency by the incorporation of a Warburg component in the circuit, and the depressed semicircle by a constant phase element (CPE), while resistors Rs reproduces the bulk resistance. The low values for Rs (0.606 Ω, CNT-Fe, and 0.846 Ω, CNT-Fe + PPy) compared to much higher PPy value (46.23 Ω) confirm that both SCs electrodes preserve low bulk resistance with added small resistance attributed to the addition of Fe nanoparticles. However, these results confirm that both CNT-Fe and CNT-Fe + PPy electrodes have favorable kinetics for ion diffusion. The lower Rs could be caused by the synergistic effect of CNT and iron nanoparticles, which can effectively shorten the electron transportation pathway and improve electrical conductivity and charge transportation kinetic of the electrodes^66^.

The capacitive behavior of the CNT-Fe, CNT-Fe + PPy, and PPy materials in SCs systems was further evaluated through their frequency analysis^67,68^ (see Fig. 7c,d, respectively). Figure 7c, (for the real part of the capacitance—C′) shows the variation of the available stored energy with the frequency. As can be observed, the capacitance of the three-based SCs is available at long frequency intervals (from lower to higher). The CNT-Fe + PPy presented the highest capacitance among all but only at the narrow frequency interval near 10^–1^ Hz, while CNT-Fe exhibited the highest capacitance at the interval of 0.3 to 100 Hz. In addition, the typical behavior for the real part of capacitance was observed for both experimental systems with an increase from almost zero at higher frequencies to a maximum value at lower frequencies—characterizing a typical transition from resistive to capacitive behavior confirmed from the peak in the imaginary part of the capacitance.

### Electrical performance of CNT-Fe and CNT-Fe + PPy based triboelectric nanogenerator

**Figure 8:**
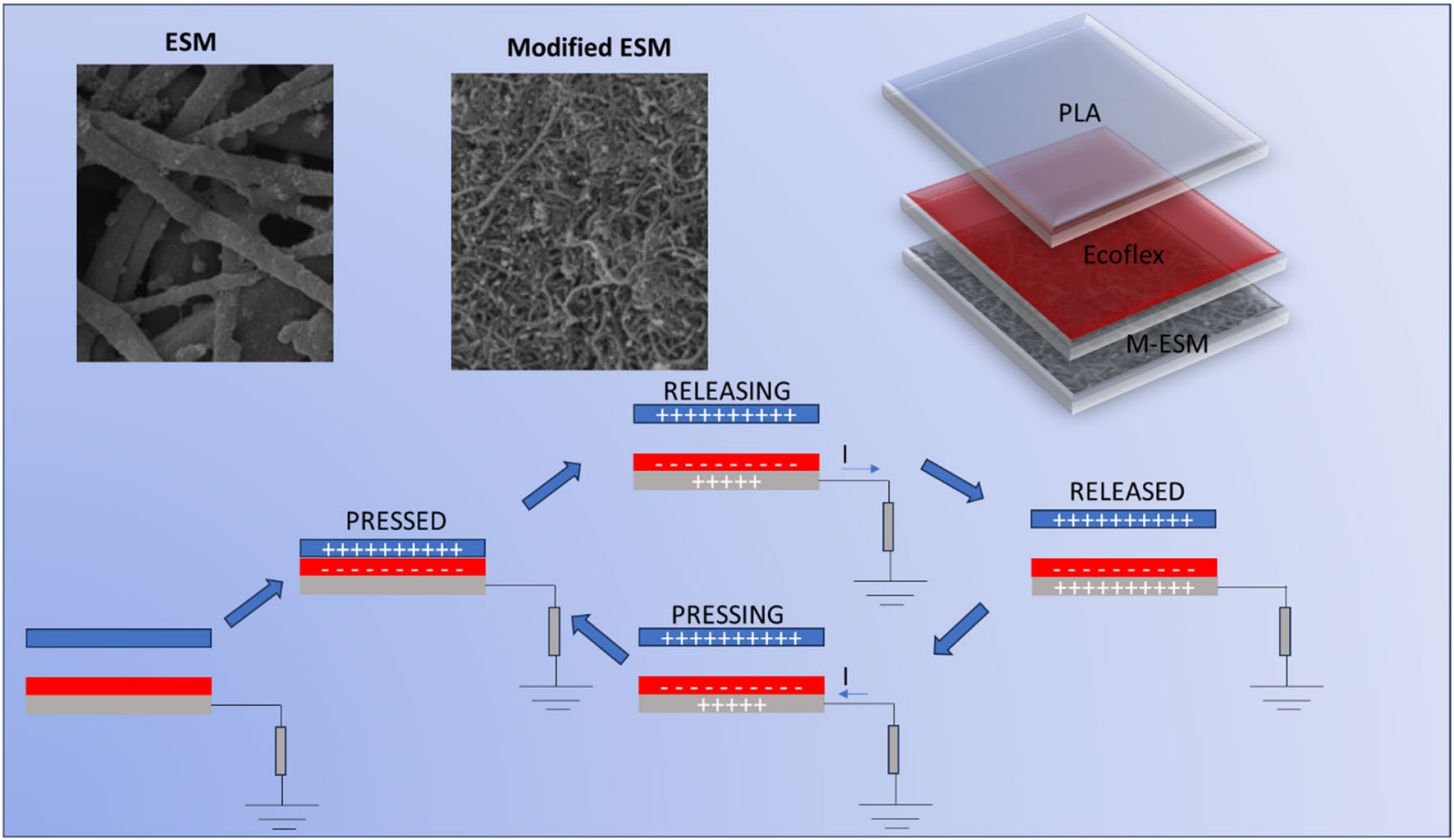
Scheme for device assembly and proposed working mechanism for the charge accumulation and transference to load resistance under cyclic contact-separation process.

Modified eggshell membranes were also applied as electrodes of triboelectric nanogenerators, that explore the combination of contact electrification (tribopositive and tribonegative layers) and electrostatic induction processes that separate positive and negative charges under contact resulting in an electric field under release/compressing forces. The charge circulation along with the electrode is mutually dependent on the conductivity of the electrode and the rugosity of the resulting friction layers that are kept in contact with the tribonegative component. The assembled device schematically shown in Fig. 8 is composed of a moving 3D printed layer of polylactic acid (PLA) of 4 cm × 4 cm and a layer of Ecoflex™ of 2 cm × 3 cm that is disposed on the electrode of ESM modified with PPy, Fe + CNT, and CNT-Fe + PPy, respectively. Device operating parameters are 7 Hz frequency, 4.5 N load, and 3 cm separation distance.

As shown, the proposed mechanism is composed of a cyclic sequence of steps in which the tribopairs are contacted and separated. In the first step, in which the layers are contacted one against the other, charges accumulate on the opposite sides of electrodes and create a resulting electric field under the release of the pressing force. This release contributes to the circulation of electrical charges in the direction of the load resistance. At the condition of the complete release of this force, the current in the load resistance tends to zero and returns in the opposite direction under pressing, providing the behavior of an alternating current generator with a cycle defined at the end of the complete compression step of layers. Based on these constitutive aspects, the triboelectric nanogenerators produced at different conditions for surface modification were tested at different frequencies of operation.

**Figure 9:**
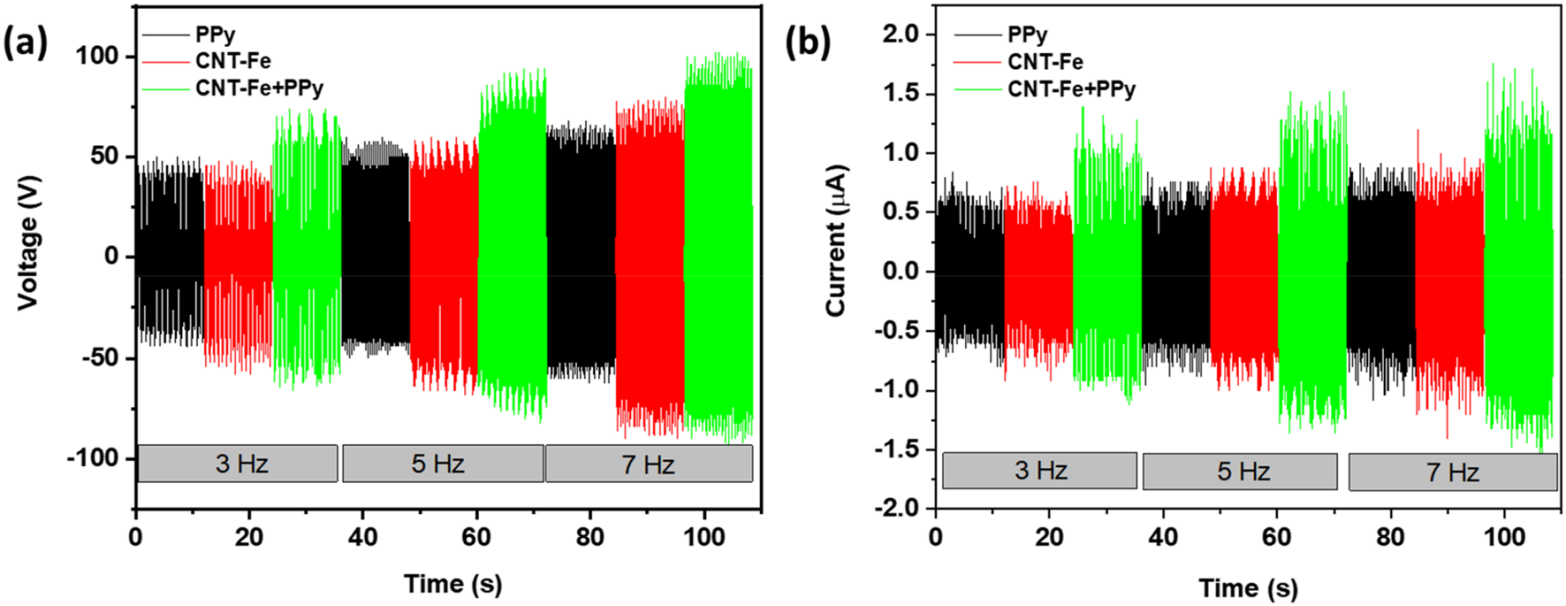
(**a**) Open circuit voltage at frequencies of 3 Hz, 5 Hz, and 7 Hz for samples coated with PPy, CNT-Fe, and CNT-Fe + PPy, (**b**) short circuit current at frequencies of 3 Hz, 5 Hz. and 7 Hz for samples coated with PPy, CNT-Fe, and CNT-Fe + PPy.

Results in Fig. 9a indicate that the response of open circuit voltage increases with the frequency of operation in response to the energy transfer ratio from tribopairs to the electrode. In addition, higher performance was observed for samples modified with CNT-Fe + PPy in comparison with PPy and Fe + CNT electrodes. In correspondence, the same behavior was observed in the short-circuit current (see Fig. 9b). The measurement of the medium of peaks is plotted in Figs. S4a and S4b indicate that higher voltage is observed for devices prepared with PPy + CNT-Fe with a slight increase in the values for samples modified with Fe + CNT in comparison with samples modified with PPy. The best performance observed for CNT-Fe + PPy agrees with the best conductivity observed for the electrodes incorporating CNT-Fe + PPy and the fibrilar structure of the resulting material. Based on these considerations, the best device was defined as the TENG prepared with the electrode of CNT-Fe + PPy and operated at 7 Hz. It is worth mentioning that there are two distinct regions regarding the electrical output performance of TENGs as a function of the excitation frequency. At low-frequency regions (typically for f < 7 Hz) the typical behavior is of electrical output increase with increasing excitation frequency^69^, in a process attributed to the activation of additional triboelectric charges that are not completely neutralized during the contact-separation process at higher frequencies. On the other hand, for all frequencies, the performance (for both open circuit voltage and short circuit current) follows the order CNT-Fe + PPy > CNT-Fe > PPy which agrees with the order in electrochemical performance of supercapacitors, characterizing those electrical properties as the most relevant parameter to define the performance of TENG since lower internal resistance facilitates the charge transference (for short circuit current) and offers available sites for charge accumulation improving the open circuit voltage response by the adequate accumulation of charges at interfaces.

**Figure 10:**
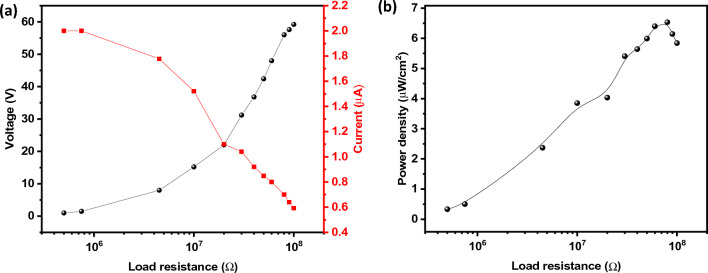
(**a**) Dependence of the output current and voltage as a function of the load resistance and (**b**) power density of the device as a function of the load resistance (curves acquired at 7 Hz for sample CNT-Fe + PPy).

For this configuration, the output performance of the device was evaluated for different load resistances. Curves in Fig. 10a for current and voltage indicate the typical crossover due to the inverse variation of functions: while the current decreased with the increasing value of the load resistance the voltage increased reaching a maximum value in the order of 60 V while a maximum current is observed at 2 µA. The power density of the device as a function of the load resistance is shown in Fig. 10b, a clear direct relationship is observed between both loading resistance and power density with a well-defined maximum power output density at 6 μW/cm^2^ (see Fig. 10b and Table 3).

**Table 3:** The performances of different electrode materials for TENGs from the literature and this work.

Electrode materials	V_OC_ (V)	I_SC_ (µA)	P (µW cm^−2^)	Ref.
Pani@WCT	350	45	1130	^70^
CNT	7	0.18	–	^71^
PPy	60	8.8	8.3	^72^
C-coffee ground	150	2.1	6.38	^73^
Organogel	40	1.2	4	^74^
Carbon fiber	42.9	0.51	0.6	^75^
Degradable microbial cellulose	1010	–	870	^76^
CNT-Fe + PPy	60	2	6	This work

**Figure 11:**
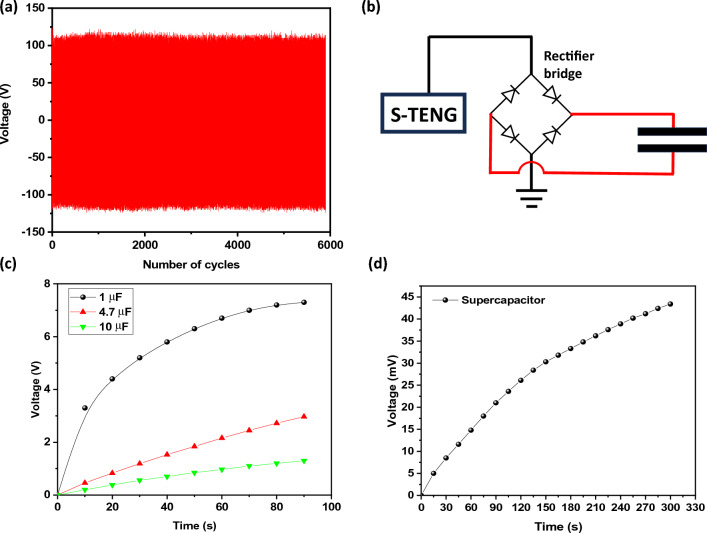
(**a**) Retention in the value of the generated voltage at successive 6000 cycles operation of the device, (**b**) circuit with a rectifier bridge applied in the charge of capacitors, (**c**) curves of charge of the capacitor of 1, 4.7, and 10 µF, and (**d**) sample CNT-Fe + PPy-based supercapacitor, disposed of in the output of the TENG (curves acquired at 7 Hz for sample CNT-Fe + PPy).

Another important factor to be considered for the prolonged use of the device is the retention of the electrical properties under repeated operation. As shown in Fig. 11a, negligible variation in the output voltage was observed indicating that prolonged operation minimally affects the performance of the device after 6000 cycles of operation. For the application of TENG as a charging element of conventional capacitors and supercapacitors, the circuit shown in Fig. 11b was implemented to provide a pulsed DC signal to charge the capacitors and supercapacitors in an integrated combination of TENG/SC.

For comparison in the performance of charging processes, the response of conventional capacitors (1, 4.7, and 10 µF) disposed of in the output of the TENG via a rectifier bridge and the supercapacitor based on the sample CNT-Fe + PPy were evaluated. Under the operation at 7 Hz, curves in Fig. 11c indicate that higher voltages are accumulated for capacitors with lower capacitance—due to the dependence of voltage V with the capacitance C (V = q/C). As observed, the greater the capacitance, the longer the time for reaching higher voltage on terminals. As expected, the corresponding curve for the supercapacitor integrated with the TENG is shown in Fig. 11d, in which a variation in the accumulated voltage of 42 mV is observed after 300 s of operation of the TENG, confirming the potential of integration between TENG and SC with the same substrate (CNT + PPy on ESM).

## Conclusions

The synthesis of advanced flexible SCs and TENGs’ electrode materials through a sustainable and greener approach using eggshell membrane as flexible support material and a plant-based reductant (*Punica Granatum*) as a reducing agent to form and deposit iron NPs on carbon nanotube structure acting as a support for electrode fabrication was successfully conducted. The eggshell membranes were coated with iron-NPs-NT material to serve as sustainable and flexible electrodes. Further, the coated eggshell membranes with iron-NPs-NTs material were subjected to a chemical polymerization with polypyrrole (PPy), which creates a layer over the flexible electrode that preserves the electrochemical properties of additives (EDLC and PC). The flexible electrodes were fully characterized in detail to evaluate the role of PPy on the physicochemical and electrochemical properties of SCs and TENGs. The physicochemical characterization data suggested a very high influence of the PPy on the CNT-Fe structure. The electrochemical data revealed that the incorporation of PPy owned better electrochemical performance due to the combination of quasi-electric double-layer capacitance behavior added to the pseudocapacitance contribution. The areal capacitance values were 7 and 202 mF/cm^2^ at 1 mA/g for CNT-Fe and CNT-Fe + PPy samples, respectively. When tested as electrodes in TENGs, they indicated performances of 60 V for output voltage and power density of 6 μW/cm^2^ for the CNT-Fe + PPy sample. The integrated system showed that the supercapacitors can be successfully charged directly by the nanogenerator from 0 to 42 mV in 300 s. The successful green synthesis of iron nanoparticles on CNT and further PPy coating provides a feasible method for the design and synthesis of high-performance SCs and TENGs electrode materials as integrated components.

### Supplementary Information

Supplementary Figures.

## Data Availability

The data that support the findings of this study are available from the corresponding author upon reasonable request.
