# Supplementary material for: Supercapacitors and triboelectric nanogenerators based on electrodes of greener iron nanoparticles/carbon nanotubes composites
Source: Sci Rep. 2024 May 21;14:11555. doi: 10.1038/s41598-024-61173-5 (PMC11109182; doi:10.1038/s41598-024-61173-5)
Supplement: Supplementary file 1 — Supplementary Figures. [file 41598_2024_61173_MOESM1_ESM.docx]

**Supercapacitors and triboelectric nanogenerators based on electrodes of greener iron nanoparticles/ carbon nanotubes composites**

Glaydson Simoes dos Reis^1,#,*^, Helinando Pequeno de Oliveira^2,#^, Iuri C.M. Candido^2^, Andre L. Freire^2^, Palanivel Molaiyan^3^, Guilherme Luiz Dotto^4^, Alejandro Grimm^1^, Jyri-Pekka Mikkola^5,6^

^1^Department of Forest Biomaterials and Technology, Biomass Technology Centre, Swedish University of Agricultural Sciences, Umeå SE-901 83, Sweden.

^2^Institute of Materials Science, Federal University of Sao Francisco Valley, Petrolina 56304-205, Brazil

^3^Research Unit of Sustainable Chemistry, University of Oulu, P.O. Box 3000, FI-90014, Oulu, Finland.

^4^Research Group on Adsorptive and Catalytic Process Engineering (ENGEPAC), Federal University of Santa Maria, Av. Roraima, 1000-7, 97105–900 Santa Maria, RS, Brazil.

^5^Technical Chemistry, Department of Chemistry, Umeå University, Umeå, 90187, Sweden

^6^Industrial Chemistry and Reaction Engineering, Johan Gadolin Process Chemistry Centre, Åbo Akademi University, Åbo-Turku, 20500, Finland

^#^These authors are equally contributed.

*Corresponding author: Glaydson Simoes dos Reis ([glaydson.simoes.dos.reis@slu.se](mailto:glaydson.simoes.dos.reis@slu.se))

## **Characterization of electrode materials**

The morphology evaluation of the biochars was carried out using a scanning electron microscope Vega 3XM (Tescan) with an electron acceleration of 10 kV, with the collected images collected with magnifications of 500 x, 1 kx, 3 kx, and 5 kx. The SSA was calculated from Brunauer–Emmett–Teller (BET) method, and pore size distribution using the Barrett−Joyner−Halenda (BJH) model.

Functional group determination was done using a Fourier Transform Infra-Red (FTIR) spectrometer (Bruker, model alpha) in the range 4000–400 cm^−1^, using pellets with 0.01 g of samples dispersed in 0.2 g of KBr.

The nature of solid phases in the samples was analyzed using a powder X-ray diffraction instrument (Siemens). XRD patterns were obtained with a Diffrac 5000 BRUKER diffractometer (USA) operating at 45 kV and 40 mA using Cu-Kα monochromatic radiation (λ = 1.54 Å), 2θ angle interval of 2–80° with counting time of 5 s/pass, step size of 0.05° in 2θ, and slit widths of 200 μm. The instrument was operated under certain controlled and fixed conditions.


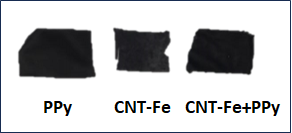


**Figure S1.** Images of electrodes based on the modified eggshell membrane: PPy, CNT-Fe, and CNT-Fe+PPy


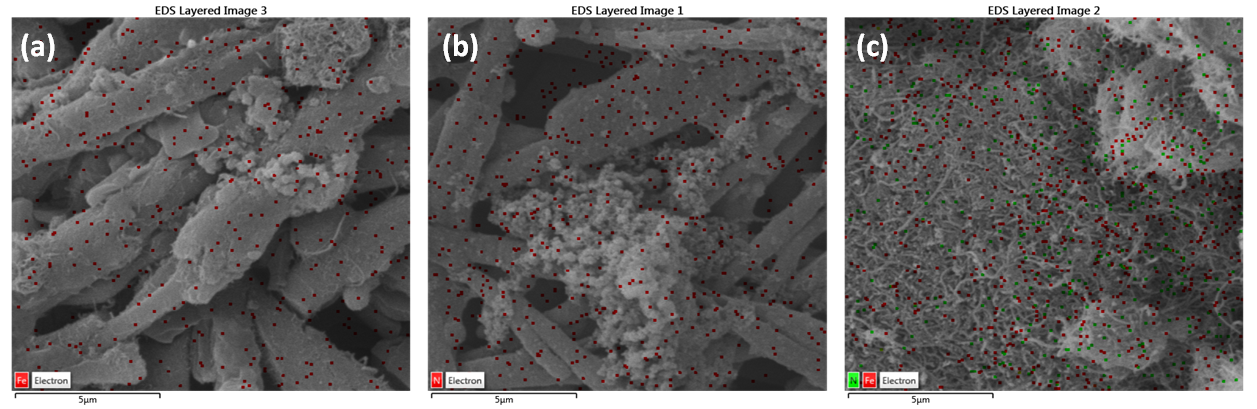


**Figure S2.** Overlaid EDS images (red dots for Fe element and green dots for N element).


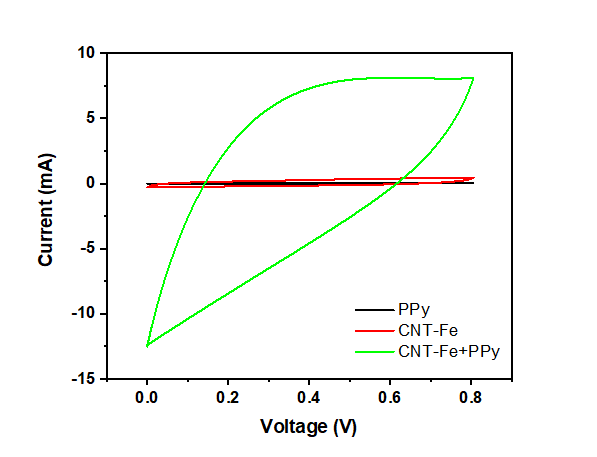


**Figure S3.** Comparison of CV curves for different electrodes at 100 mVs^-1^.


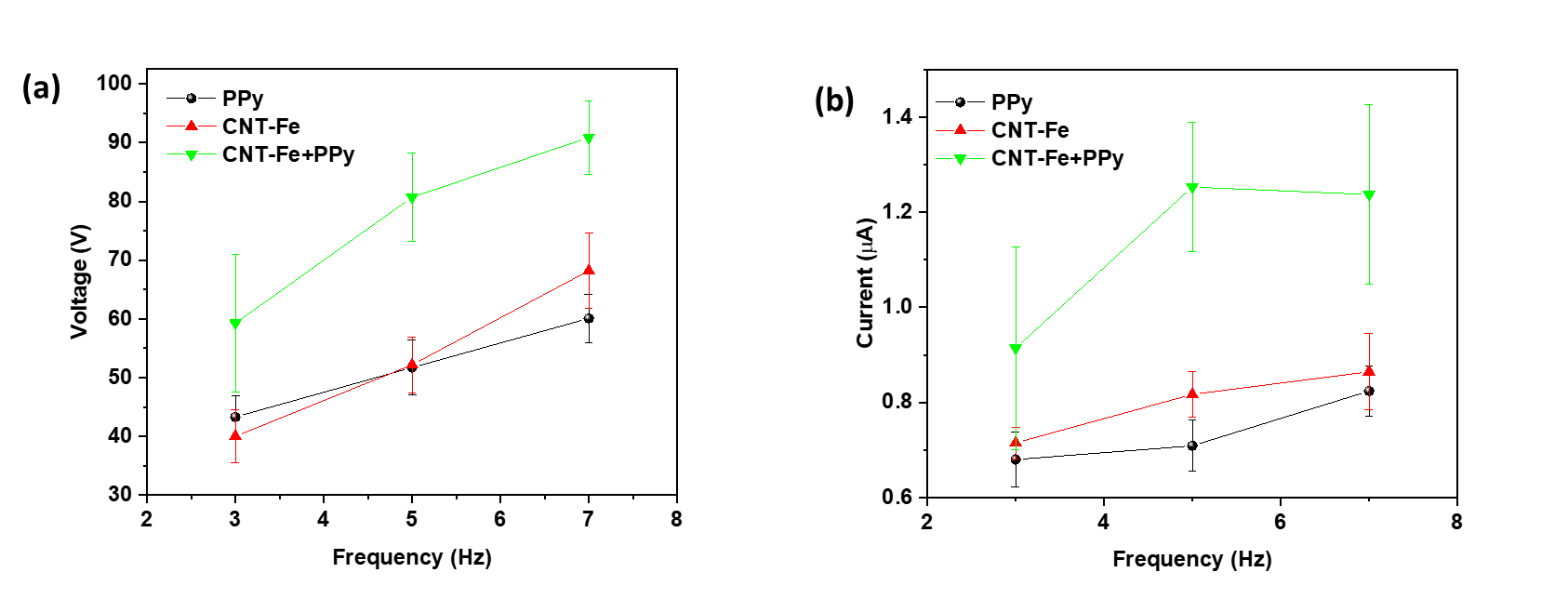


**Figure S4.** (a) Medium of peaks of V_OC_ for samples coated with PPy, CNT+Fe, and CNT+Fe-PPy, and (b) medium of peaks of I_SC_ for samples coated with PPy, CNT+Fe, and CNT+Fe-PPy.
